# Supplementary material for: The Importance of Epigallocatechin as a Scaffold for Drug Development against Flaviviruses
Source: Pharmaceutics. 2023 Mar 1;15(3):803. doi: 10.3390/pharmaceutics15030803 (PMC10053286; doi:10.3390/pharmaceutics15030803)
Supplement: Supplementary file 1 [file pharmaceutics-15-00803-s001.zip › pharmaceutics-2208239-supplementary.pdf]

Supplementary Information

The Importance of Epigallocatechin as a Scaffold for Drug Development against Flaviviruses

Mônika A. Coronado [1,*], Ian Gering [1], Marc Sevenich [1,2], Danilo S. Olivier [3], Mohammadamin Mastalipour [1,4], Marcos S. Amaral [5], Dieter Willbold [1,4,6] and Raphael J. Eberle [1,4,*]

1 Institute of Biological Information Processing (IBI-7: Structural Biochemistry), Forschungszentrum Jülich, 52428 Jülich, Germany
2 Priavoid GmbH, Merowingerplatz 1A, 40225 Düsseldorf, Germany
3 Integrated Sciences Center, Campus Cimba, Federal University of Tocantins, Araguaína 77824-838, TO, Brazil
4 Institut für Physikalische Biologie, Heinrich-Heine-Universität Düsseldorf, Universitätsstraße, 40225 Düsseldorf, Germany
5 Institute of Physics, Federal University of Mato Grosso do Sul, Campo Grande 79070-900, MS, Brazil
6 JuStruct: Jülich Centre for Structural Biology, Forschungszentrum Jülich, 52428 Jülich, Germany
* Correspondence: m.coronado@fz-juelich.de (M.A.C.); r.eberle@fz-juelich.de (R.J.E.); Tel.: +49-2461-61-9505 (M.A.C. & R.J.E.)

Table of contents

Table S1. Range of the epigallocatechin molecules concentrations.

Figure S1. Inhibition effect of the Epigallocatechin molecules over the NS2B/NS3 proteases.

Figure S2. IC_{50} calculation of the Epigallocatechin molecules over the NS2B/NS3 proteases.

Figure S3. Inhibition mode of the Epigallocatechin molecules over the NS2B/NS3 proteases.

Figure S4. Inhibition effect of the Epigallocatechin molecules over the NS2B/NS3 proteases.

Figure S5. Dissociation constant (K_D) determination of the epigallocatechin molecules binding to $NS2B/NS3^{pros}$ using surface plasmon resonance (SPR).

Figure S6. Ligand-iduced changes in the CD spectrum of soluble NS2B/NS3 proteases.

Figure S7. Fluorescence spectroscopy of Trp at 295 nm of the $NS2B/NS3^{pros}$ and the epigallocatechin molecules.

Figure S8. RMSD values and RMSF profiles for the replicate 100 ns of MD simulations of NS2B/NS3 protease of DENV in complex with the epigallocatechin molecules.

Figure S9. RMSD values and RMSF profiles for the replicate 100 ns of MD simulations of NS2B/NS3 protease of YFV in complex with the epigallocatechin molecules.

Figure S10. RMSD values and RMSF profiles for the replicate 100 ns of MD simulations of NS2B/NS3 protease of WNV in complex with the epigallocatechin molecules.

Figure S11. RMSD values and RMSF profiles for the replicate 100 ns of MD simulations of NS2B/NS3 protease of ZIKV in complex with the epigallocatechin molecules.

Figure S12. Numbers of H-bonds between the epigallocatechins and NS2B/NS3pro residues during the 100 ns MD simulations.

Table S1. Range of the epigallocatechin molecules concentrations.

Epigallocatechin molecules concentration (µM)			
	EGC	EGCG	AcEGCG

**DENV2**	1.31; 1.97; 2.96; 4.44	0.58; 0.88; 1.32; 1.97	1.95; 2.9; 4.38; 9.87; 14.81
**YFV**	1.75; 2.63; 3.95; 5.92; 8.88; 13.33	0.39; 0.58; 1.31; 1.97; 2.9; 4.44	1.97; 2.9; 4.44; 6.66; 10
**WNV**	1.56; 3.12; 6.25; 12.5	1.3; 1.95; 2.9; 4.38; 6.5	4.38; 6.5; 9.9; 14.81; 22.2
**ZIKV**	4; 8; 16; 32; 64	0.58; 0.88; 1.32; 1.97	2.92; 4.39; 6.58; 9.87; 14.81; 22.22; 33.33

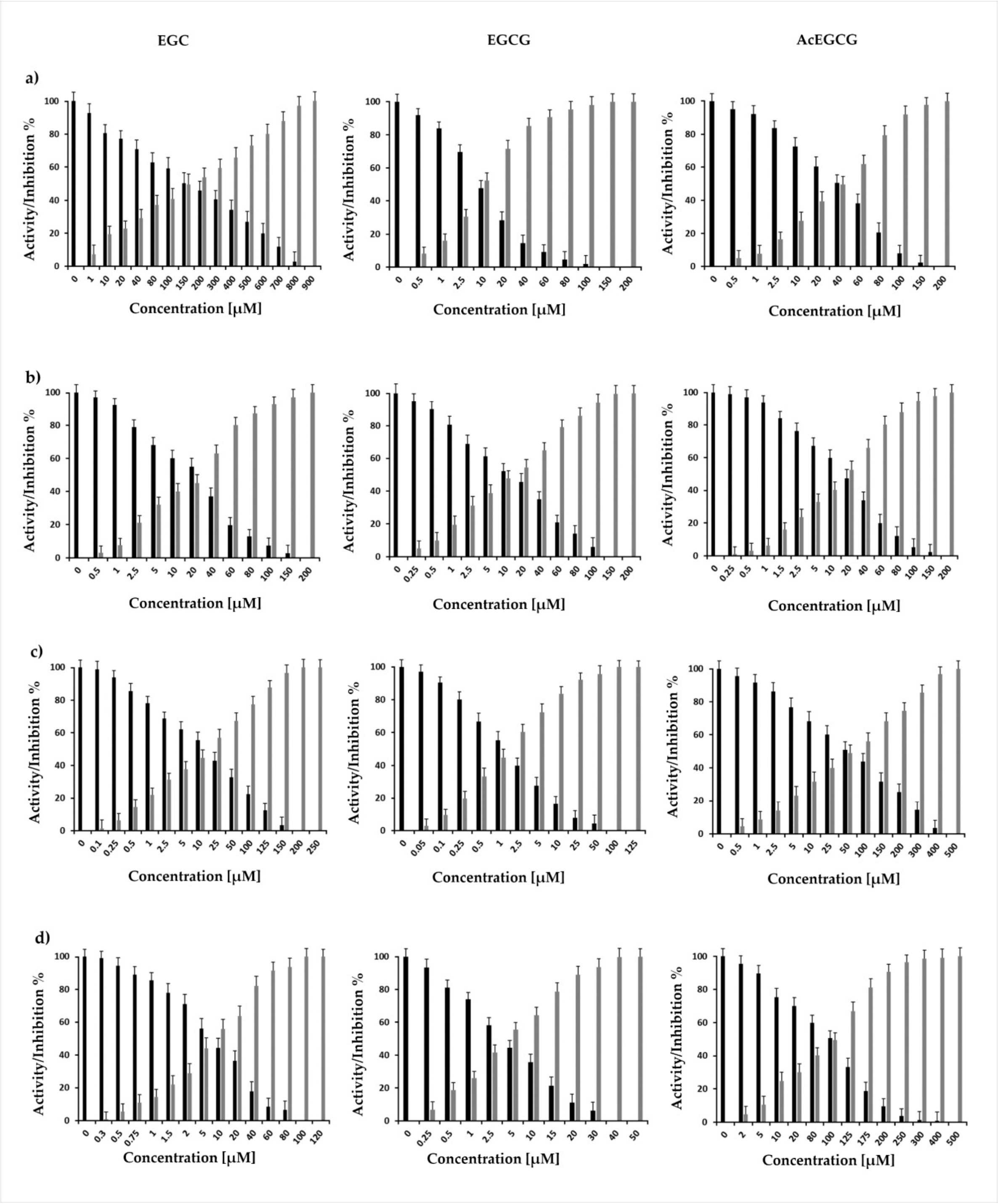

Figure S1. Inhibition effect of the Epigallocatechin molecules over the NS2B/NS3 proteases of (a) DENV, (b) YFV), (c) WNV, and (d) ZIKV. Data showed are the mean ± SD from three independent measurements (n=3).

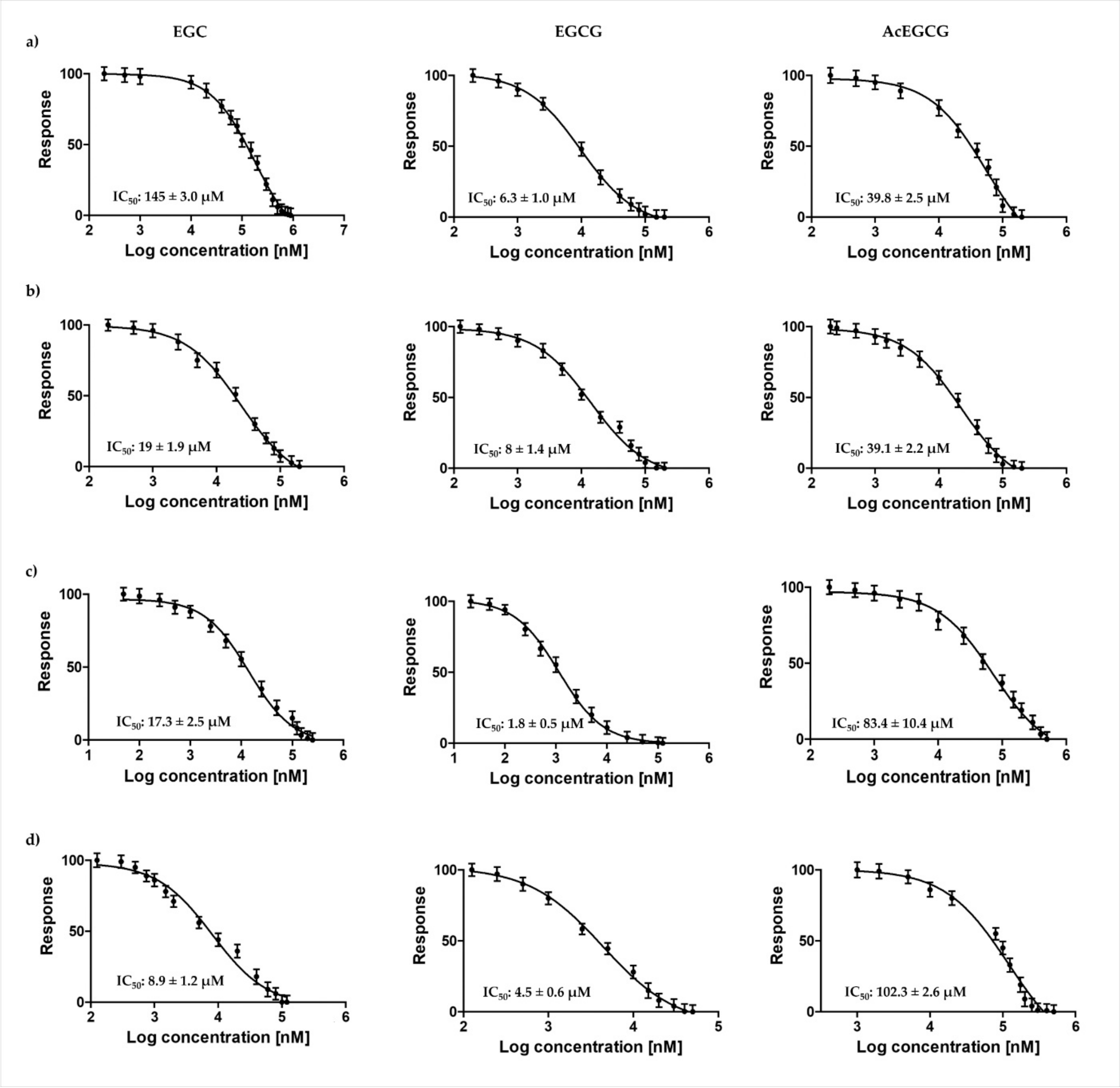

Figure S2. IC_{50} calculation of the Epigallocatechin molecules over the NS2B/NS3 proteases of (a) DENV, (b) YFV), (c) WNV, and (d) ZIKV. Data showed are the mean ± SD from three independent measurements (n=3).

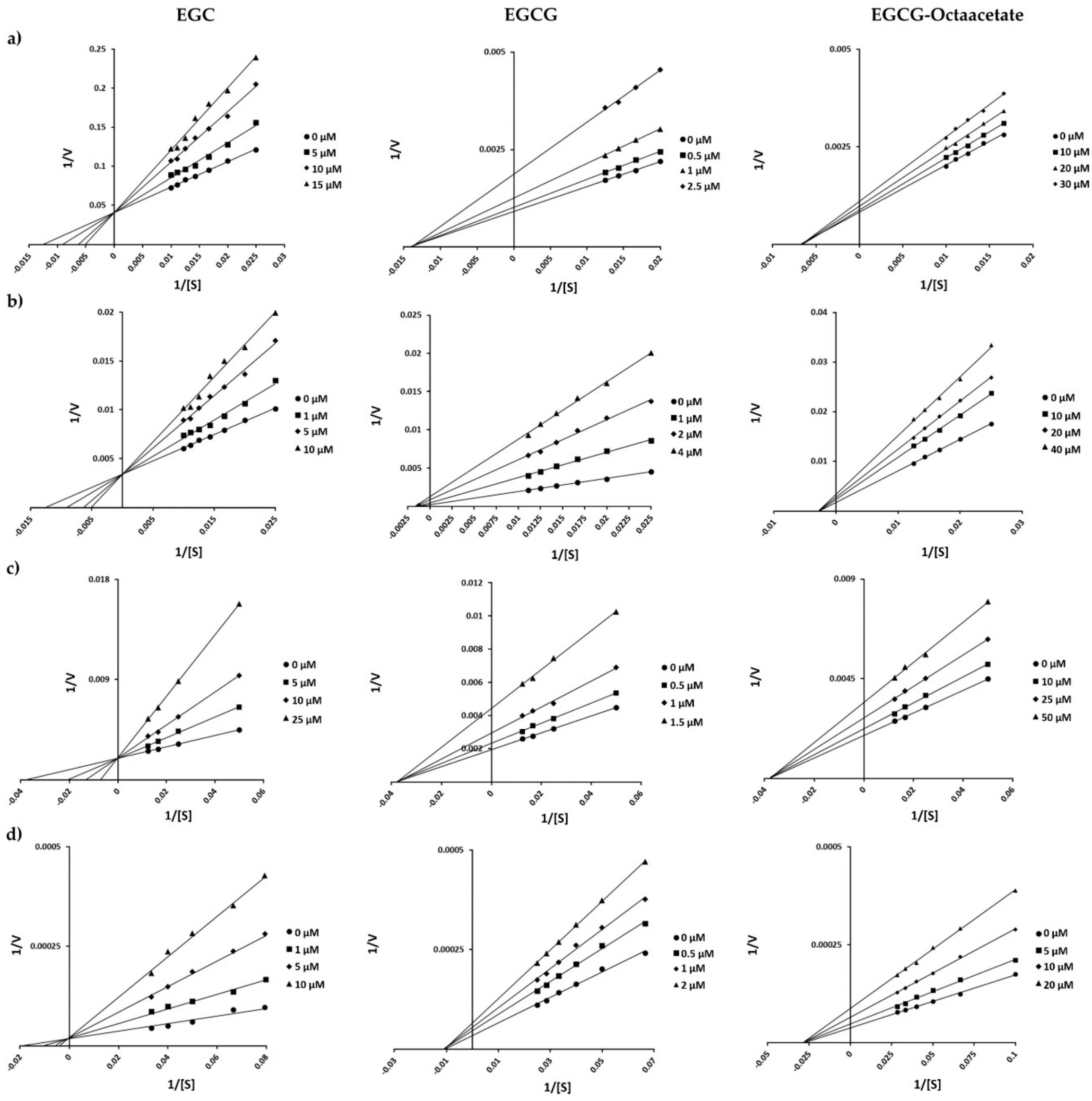

Figure S3. Inhibition mode of the Epigallocatechin molecules over the NS2B/NS3 proteases of (a) DENV, (b) YFV), (c) WNV, and (d) ZIKV. Data showed are the mean ± SD from three independent measurements (n=3).

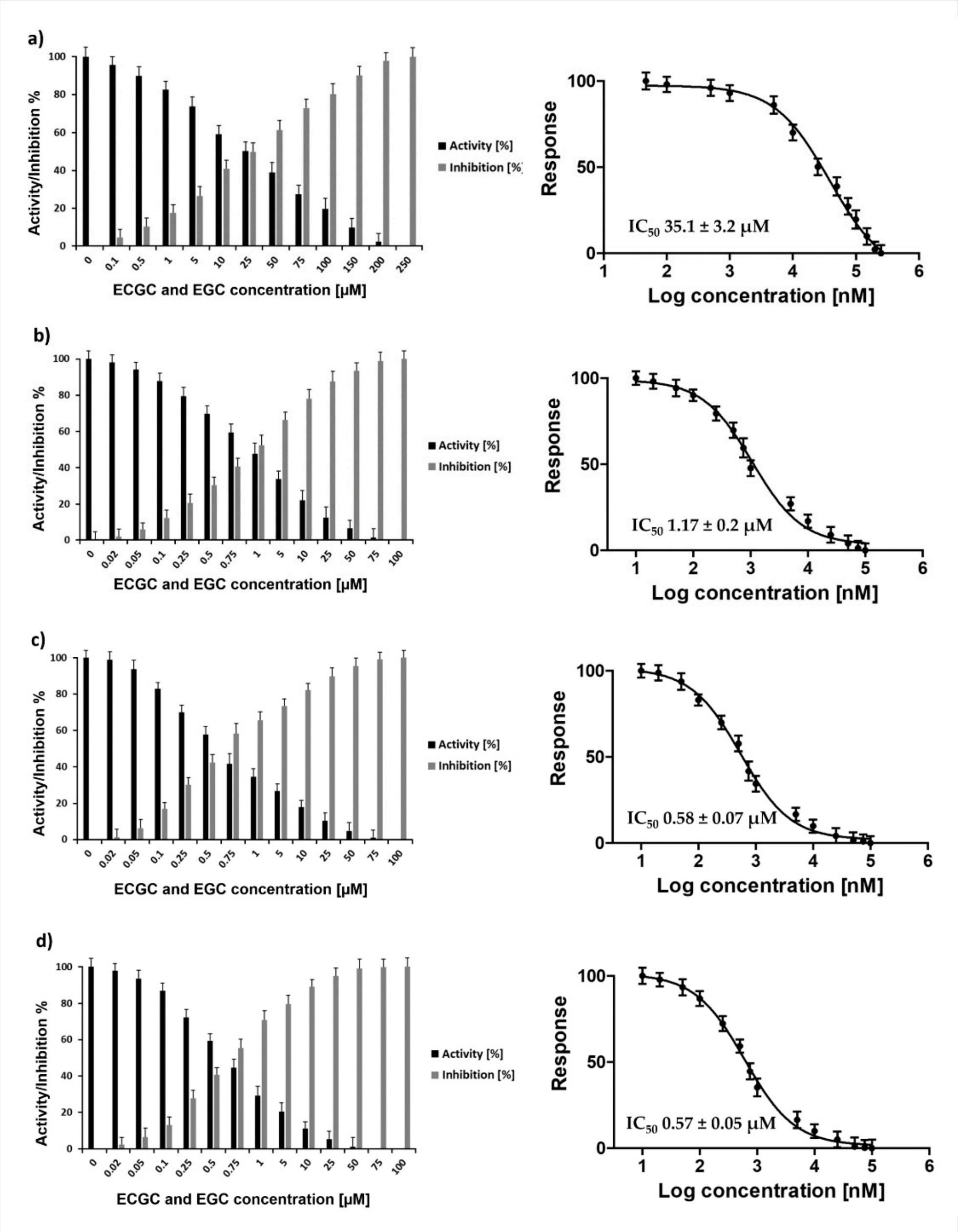

Figure S4. Inhibition effect of the Epigallocatechin molecules combination over the NS2B/NS3 protease of (a) DENV, (b) YFV), (c) WNV, and (d) ZIKV. Data showed are the mean ± SD from three independent measurements (n=3).

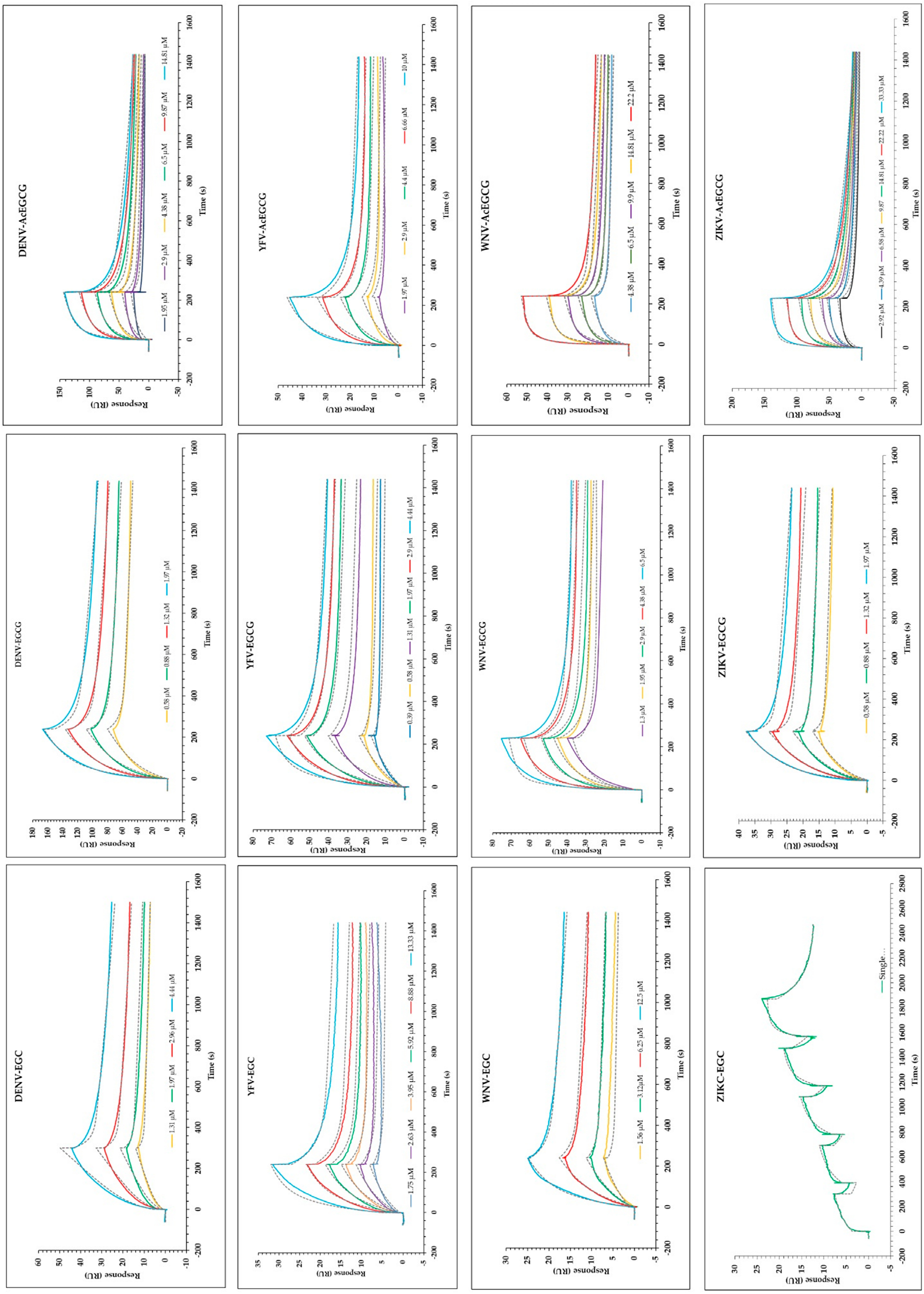

Figure S5. Dissociation constant (KD) determination of the epigallocatechin molecules binding to NS2B/NS3^{pros} using surface plasmon resonance (SPR). NS2B/NS3 proteases were immobilized on CM5 sensor chip and a range of the epigallocatechin molecules concentrations (Table S1) were passed over the surface. The final sensogram is representative of two cycles.

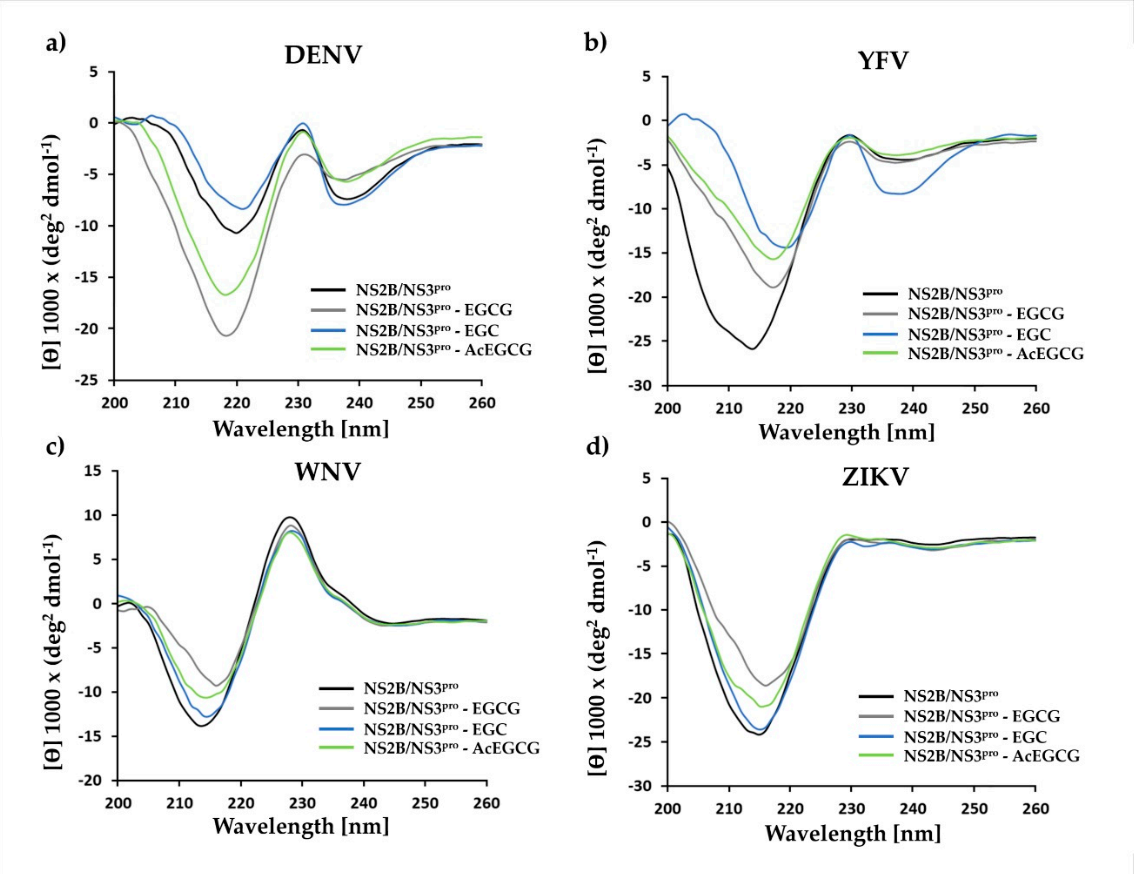

Figure S6. Ligand-iduced changes in the CD spectrum of soluble NS2B/NS3 proteases of DENV, YFV, WNV and ZIKV in 20 mM K2HPO4/KH2PO4 pH 7.5.

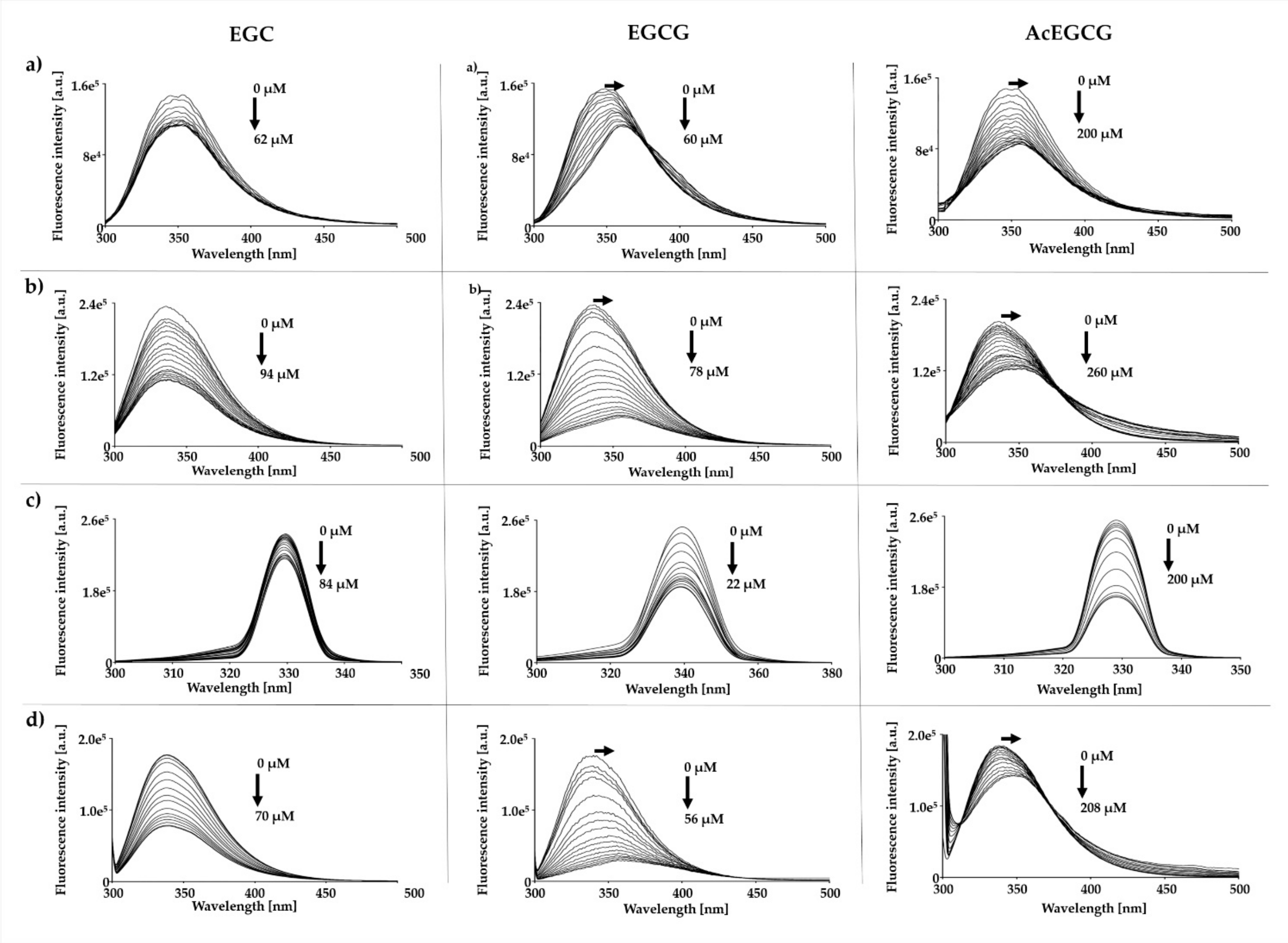

Figure S7. Fluorescence spectroscopy of Trp at 295 nm of the NS2B/NS3pros and the epigallocatechin molecules. a) DENV, (b) YFV), (c) WNV, and (d) ZIKV.

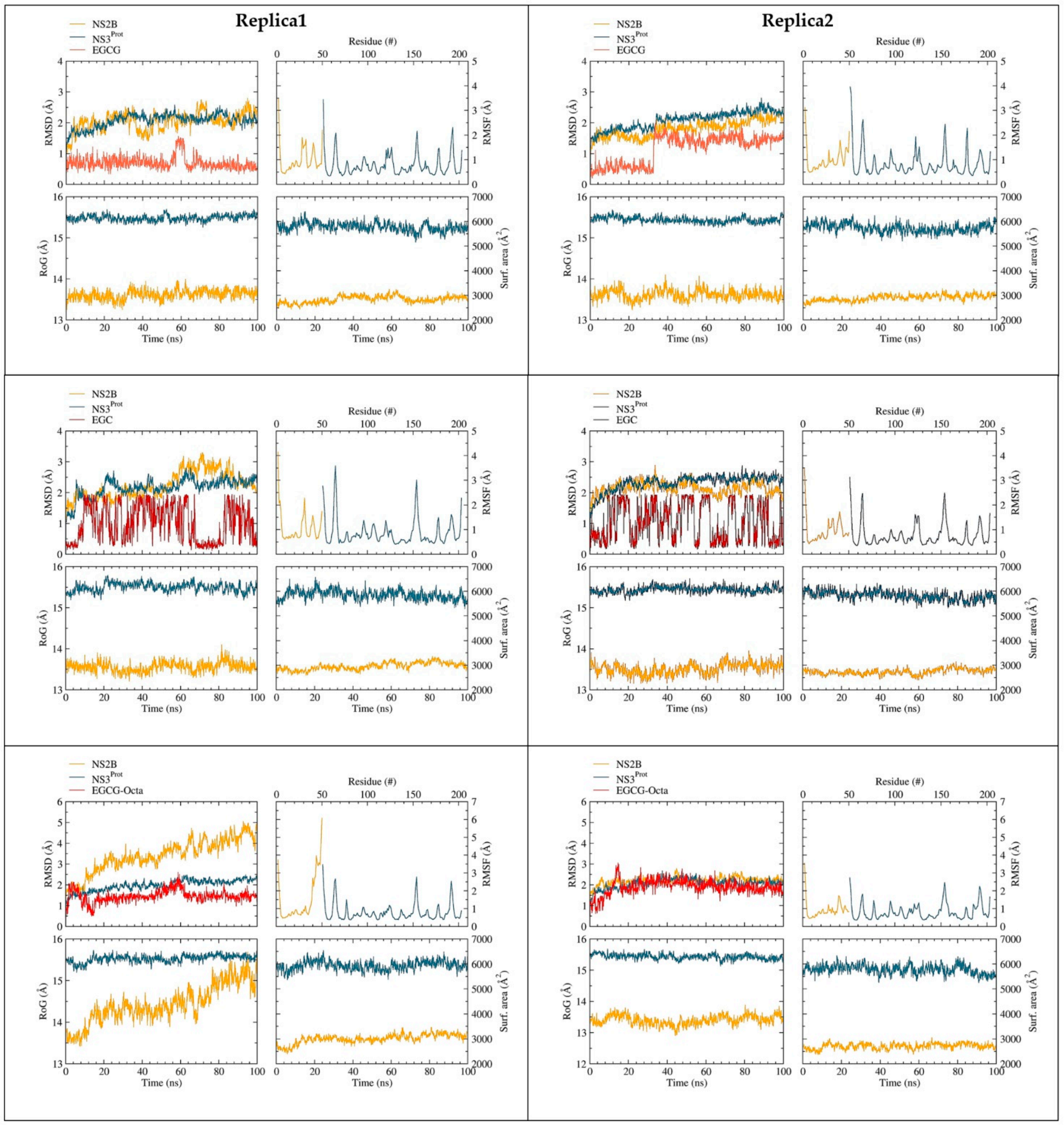

Figure S8 RMSD values and RMSF profiles for the replicate 100 ns of MD simulations of NS2B/NS3 protease of DENV in complex with the epigallocatechin molecules. The RMSD time profiles concerning all backbone atoms of NS2B/NS3^{pros} and the molecules (EGC, EGCG, and AcEGCG). The RMSF plot corresponds to the protein/molecules complex, and the replicates are specified.

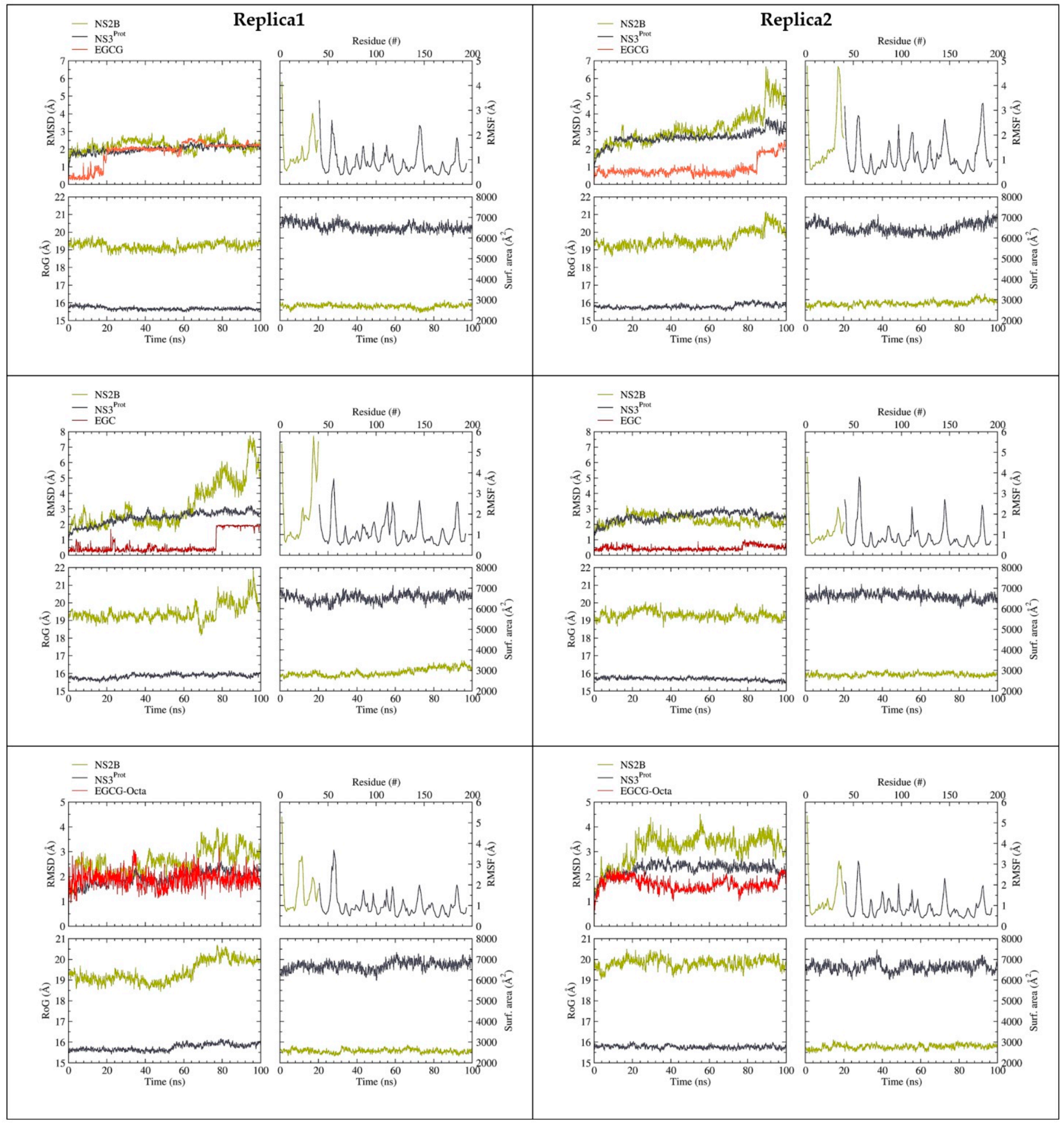

Figure S9. RMSD values and RMSF profiles for the replicate 100 ns of MD simulations of NS2B/NS3 protease of YFV in complex with the epigallocatechin molecules. The RMSD time profiles concerning all backbone atoms of NS2B/NS3^{pros} and the molecules (EGC, EGCG, and AcEGCG). The RMSF plot corresponds to the protein/molecules complex, and the replicates are specified.

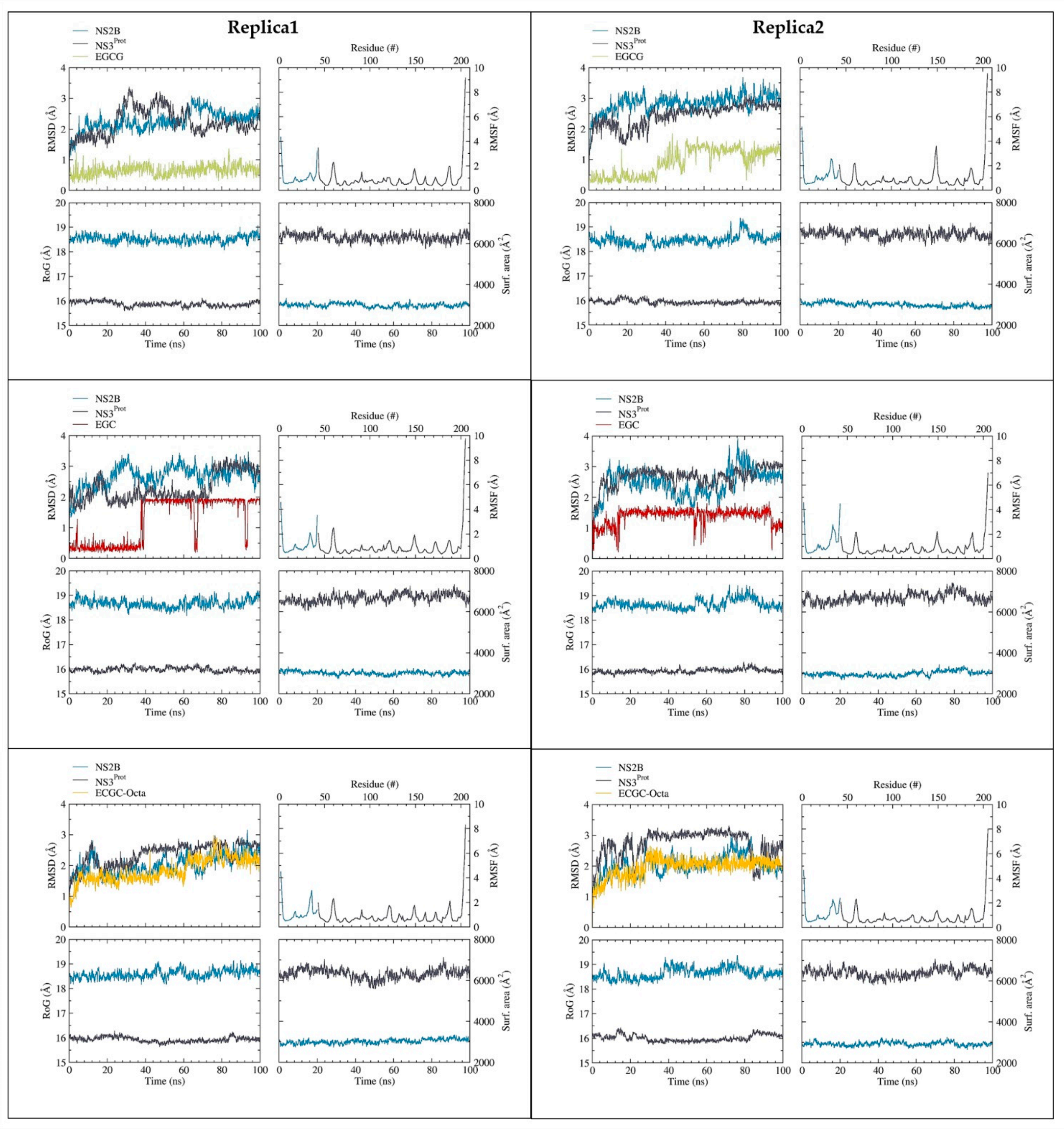

Figure S10. RMSD values and RMSF profiles for the replicate 100 ns of MD simulations of NS2B/NS3 protease of WNV in complex with the epigallocatechin molecules. The RMSD time profiles concerning all backbone atoms of NS2B/NS3^{pros} and the molecules (EGC, EGCG, and AcEGCG). The RMSF plot corresponds to the protein/molecules complex, and the replicates are specified.

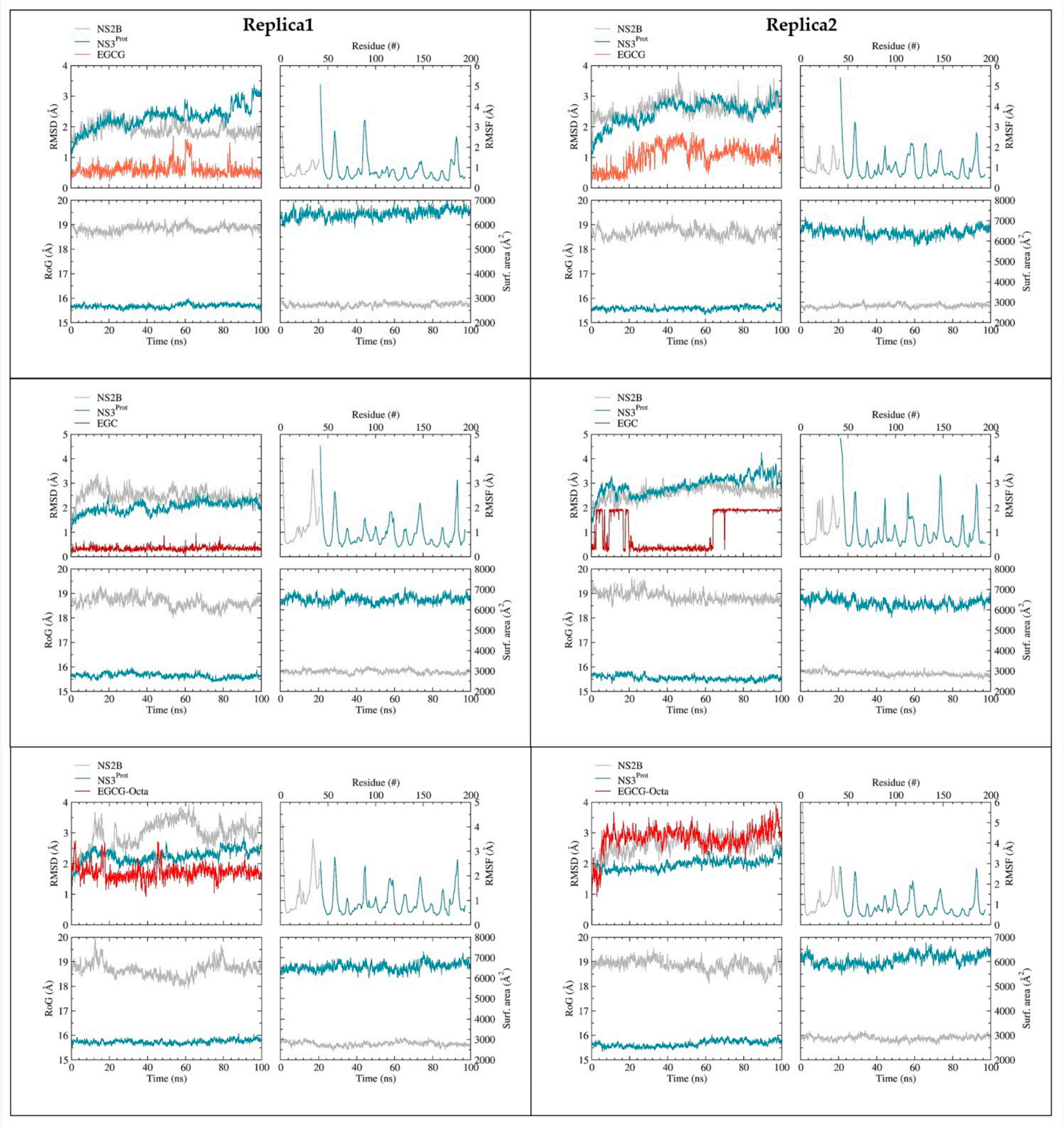

Figure S11. RMSD values and RMSF profiles for the replicate 100 ns of MD simulations of NS2B/NS3 protease of ZIKV in complex with the epigallocatechin molecules. The RMSD time profiles concerning all backbone atoms of NS2B/NS3^{pros} and the molecules (EGC, EGCG, and AcEGCG). The RMSF plot corresponds to the protein/molecules complex, and the replicates are specified.

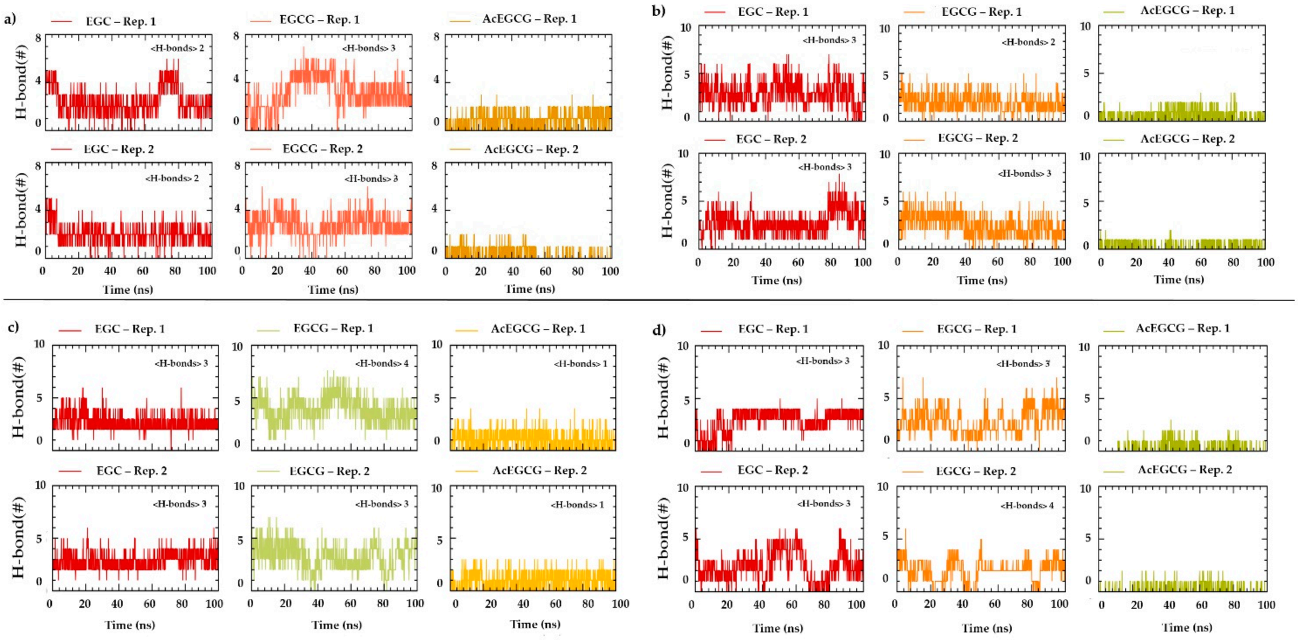

Figure S12. Numbers of H-bonds between the epigallocatechins and NS2B/NS3pro residues during the 100 ns MD simulations. The general criteria to determine the occurrence of the H-bond was a donor-receptor distance ≤ 3.5 Å and a donor-H-acceptor angle ≥ 120°. <H-donds> represents the average number of intermolecular H-bonds formed algong each trajectory. a) Dengue, b) YFV, c) WNV, and d) ZIKV.
